# On the (in)efficiency of cryptocurrencies: have they taken daily or weekly random walks?

**DOI:** 10.1016/j.heliyon.2021.e06685

**Published:** 2021-04-06

**Authors:** Natalya Apopo, Andrew Phiri

**Affiliations:** Department of Economics, Faculty of Business and Economic Studies, Nelson Mandela University, Port Elizabeth, 6031, South Africa

**Keywords:** Efficient market hypothesis (EMH), Cryptocurrencies, Random walk model (RWM), Flexible fourier form (FFF) unit root tests, Smooth structural breaks

## Abstract

The legitimacy of virtual currencies as an alternative form of monetary exchange has been the centre of an ongoing heated debated since the catastrophic global financial meltdown of 2007–2008. Our study tests the informational market efficiency of cryptomarkets by investigating the weak-form efficiency of the top-five cryptocurrencies using random walk testing procedures which are robust to asymmetries and unobserved smooth structural breaks. Moreover, our study employs two frequencies of cryptocurrency returns, one corresponding to daily returns and the other to weekly returns. Our findings validate the random walk hypothesis for daily series hence validating the weak-form efficiency for daily returns. On the other hand, weekly returns are observed to be stationary processes which is evidence against weak-form efficiency for weekly returns. Overall, our study has important implications for market participants within cryptocurrency markets.

## 1 Introduction

The role of cryptocurrencies as an alternative decentralized monetary system has gained a lot of traction following the disastrous sub-prime financial crisis of 2007–2008 which retarded the global financial system at levels not experienced since the infamous ‘Great Recession’ period of the 1930's. The digital currency market, though relatively new, is gaining increasing popularity as a ‘decentralized’ monetary system which could replace traditional centralized monetary systems and is attracting capital in lieu of the innovative technology underpinning cryptocurrencies. Proponents of digital currencies argue that the development of virtual currency markets will result in more inclusive financial markets for the future whereas opponents propose that the cryptocurrency market can only be characterised as a speculative bubble (Sovbetov, 2018). Nevertheless, the economic background of competitive private currencies such as digital currencies, though theoretically compelling, is still limited in its practical implementation. The widespread minting of virtual currencies has caused a substantial debate concerning the need for their existence, where their value is derived and if adopted, how sustainable a decentralised monetary system would look like. In any case, the sustainability of either centralised or decentralised monetary regimes, would depend on the efficiency of these monetary systems in directing the currency in circulation to its most productive uses. Owing to the fact that the technical feasibility studies of decentralised monetary systems are still at nascent stages of development, the primary focus of our study is centred on the informational efficiency of digital currency asset markets.

The role of blockchain technology in reshaping traditional financial markets as well as the innovative potential of this technology in solving some of the recurrent socio-economic problems, particularly in developing countries characterized by unstable monetary and fiscal policies, financial exclusion, extreme poverty and high corruption levels, cannot be understated (Weber, 2016). However, this innovation can only be enhanced by mitigating the risks that come with it since digital currency markets are rife with speculation. Despite the volatile nature of the cryptocurrency market as well as the risks involved in the digital currency trade, the market is growing at a rapid rate and investors as well as digital currency miners are making huge profits. It is due to these abnormally high profits, particularly those experienced in 2017, that market efficiency became a vital point of investigation. The efficiency of capital markets, of which virtual currencies have been adopted into as speculative investments, pre-empts the allocation of national resources to their most productive uses as perceived by investors. At the outset of this study, the credence of analysing the efficiency of this relatively unfettered sub-set capital markets is put forward not only because efficiency affects investment policies adopted by firms but also because it affects investing decisions taken by individual market participants. Our main concern is that speculative asset prices tend to exhibit bubble-like dynamics that eventually lead to catastrophic crashes. Therefore, evidence of market inefficiency in cryptocurrency markets would create a need for regulatory intervention in these markets and would discredit it's immediate acceptance as an alternative monetary system.

Our analytical approach is centred on informational efficiency, particularly, the weak form of the efficient markets theory in finance which is grounded in the random walk hypothesis of Nobel laureates Paul Samuelson and Eugene Fama. Our study contributes to the existing literature, Kristoufek, 2015a, Kristoufek, 2015b, Dyhberg (2016), Urquhart (2016), Bariviera et al. (2017), Kurihara and Fukushima (2017), Nadarajah and Chu (2017), Chu et al. (2017), Latif et al. (2017), Katsiampa (2017), Peng et al. (2018), Ardia (2018), Tiwari et al. (2018, 2019), Caporale and Plastun (2018), Caporale et al. (2018), Mensi (2018), Caporale and Zekokh (2019), Aggarwal (2019), Hu et al. (2019), Jana et al., 2019, Bundi and Wildi (2019), in three ways. Firstly, unlike a majority if previous studies which tend to focus on singular cryptocurrencies such as Bitcoin (see Bouri et al. (2019); Bouoiyour and Selmi (2016); Bariviera et al. (2017); Nadarajah and Chu (2017); Troster (2018); Tiwari et al. (2018); Caporale and Plastun (2018); Alvarez-Ramirez et al. (2018); Aggarwal (2019)), our study examines market efficiency in 5 cryptocurrency markets (i.e. Bitcoin (BTC), Ethereum (ETH), Litecoin, Bitcoin Cash (BTCC) and Ripple (RIP)). To the best of our knowledge, only the recent studies of Caporale et al. (2018), Brauneis and Mestel (2018), Vidal-Tomas et al. (2019), Hu et al. (2019), Kristoufek and Vosvrda (2019), Tran and Leirvik (2019), Palamalai et al. (2020) have analysed efficiency on multiple cryptocurrency. Our study contributes this relatively small but growing strand of literature. Secondly, our study employs flexible Fourier form (FFF) unit root testing procedure described in Enders and Lee (2012) which is robust to asymmetries and unobserved structural breaks which are likely to exist in high frequency financial series such cryptocurrency prices and returns. The FFF-based unit root tests are considered superior testing procedures since the econometrician does not need to know a prior the form of nonlinearity underlying the time series or the number of structural breaks which exist in the data. Thirdly, instead of focusing on one frequency of time series, for instance on daily series as is the case in an overwhelming majority of existing empirical works (see Bouoiyour and Selmi (2016), Nadarajah and Chu (2017), Latif et al. (2017), Caporale et al. (2018), Hu et al. (2019) and Aggarwal (2019)), our study employs both weekly and daily series to increase the robustness of our empirical analysis. As earlier argued by Martikainen et al. (1994) and Huang and Hoje (1995), if the underlying statistical properties for alternative return intervals for asset prices significantly differs from each other, then the efficiency of stock markets is contingent on the frequency (of) trading strategies employed by market participants. Overall, our empirical analysis presents a robust analysis of weak-form informational efficiency for the cryptocurrency market which has important implications for policymakers and investors alike.

The remainder of our study is organized as follows. The following section of the paper provides an overview of cryptocurrencies as a decentralized monetary system. The third section presents a review of the associated literature. The fourth section outlines the methodology of the study, starting with the underlying random walk model of asset returns and building onto our specific nonlinear and Fourier based unit root testing procedures. The empirical results are presented in the fifth section of the paper whilst the study is concluded in the sixth section.

## 2 Cryptocurrencies as a decentralized monetary system

The legitimacy of centralized monetary systems has been long called into question and the most recent financial crisis of 2007–2009 has further weakened the public's trust in the ability of Central Banks to manage fiat currency particularly under fractional reserve systems (Weber, 2016). The rise of Blockchain technology is envisioned as a libertarian response to the global centralized monetary systems and blockchain-based currencies employ crypto-technology to directly eliminate the need for an intermediary, such as a Central Bank, in transferring value amongst market participants. These blockchain networks facilitate inexpensive and expedient cross-border value exchanges that virtually eliminates arbitrage costs in international transactions. Digital currencies particularly offer an alternative way of managing payments in economies where Central Banks have either dismal or insufficient capital controls. And even beyond their role of currency creation, an important facet of cryptocurrencies is their decentralised public ledger network which offers more utility to market participants and is a driving factor for virtual currency valuation (Wang and Vergne, 2017).

Despite their purported benefits, the adoption of synthetic currencies as money is not without its concerns. The problems associated with the practical use of cryptocurrencies within monetary economies can be described as two-fold. Firstly, high levels of volatility in prices and returns of cryptocurrencies creates much uncertainty in valuing cryptomarkets and causes cash flow problems for businesses transactions based on digital currencies, which altogether renders cryptocurrencies ill-suited as a store of value and a unit of account (Weber, 2016). Secondly, the regulation of cryptomarkets is difficult because of the unresolved money versus speculative asset debate surrounding cryptocurrencies (Kubat, 2015). Regulatory policies required to sustain cryptocurrency technologies need to be put into place to cater for future anticipated shifts attributable to the mass adoption of novel technologies (Tasca, 2016). Consequentially, if regulation structures in decentralized currency systems are able to secure price stability and market efficiency, then the direct intervention of central authorities or private monopolies in financial markets can be kept at a minimal. However, the extent to which cryptocurrencies have been found to market efficient is subject to a growing debate as will be demonstrated in the literature review presented in next section of our study.

## 3 Review of the associated literature

Notably the empirical literature on cryptocurrencies is still in its embryonic stages of development which is not surprising when considering that virtual currencies only came into existence as a post-global financial crisis phenomenon. A systematic review of the existing literature on cryptocurrencies recently provided by Corbert et al. (2019) segregates the areas of academic focus into five categories, namely i) studies on bubble dynamics ii) studies on regulation iii) studies on cybercriminality iv) studies on diversification v) studies on market efficiency. As shown in the review, most of the existing studies are focused on the price efficiency of cryptocurrencies, with a bulk majority centred on examining the weak-form market efficiency i.e. investors cannot earn abnormal profits by analysing past data. More distinct categorizations of these studies can be made based on the differing econometric techniques used to verify or invalidate weak-form efficiency in cryptocurrencies.

Urquhart (2016) was amongst the first to examine weak-form market efficiency in cryptocurrencies by employing a battery of tests (Ljung-Box, runs test, variance ratio test, wild-bootstrap AVR test, BDS test, Hurst component) to Bitcoin returns and lent support to the inefficiency of the Bitcoin market although the author found evidence of market efficiency towards the latter periods of 2016. Using similar techniques to those employed in Urquhart (2016), Nadarajah and Chu (2017) test the weak-form efficient market hypothesis using an odd integer power of the Bitcoin returns and find evidence in favour of weak-form market efficiency. Moreover, Bariviera et al. (2017) extended upon Urquhart (2016) by computing a Hurst component via detrended fluctuation analysis and found Bitcoin to be inefficient until 2013 when the returns turn efficient. Alvarez-Ramirez et al. (2018) also made use of detrended functional analysis to reveal switching efficiency with anti-persistent behaviour being found during increasing ‘bears’ markets. Jiang et al. (2018) employ an efficiency index to examine the time-varying long-memory in Bitocin and find strong persistence in the returns series implying weak-form inefficiency. Tiwari et al. (2018) test for market efficiency by constructing a market efficiency index based on time-varying Hurst exponent and find that Bitcoin has been informationally efficient since its inception with the exception of the mid-2013 and late-2016 periods. Sensoy (2019) examine time-varying permutation entropy for high frequency returns in two Bitcoin currencies and find that whilst the whilst intra-day returns have been more efficient since 2016, higher frequency returns are more informational inefficient. Selmi et al. (2018) make use of a multifractal detrend fluctuation analysis as well as the generalized Hurst component to examine weak-form efficiency in Bitocin and found that the efficiency of Bitcoin is more prominent during ‘bear’ periods and the returns series also has a more persistent long memory in short-term components.

There also exists a separate group of studies extended the which has examined for weak-form efficiency on more than one cryptocurrency. For instance, Caporale et al. (2018) investigates long-memory behaviour in 4 cryptocurrencies returns (Bitocin, Litecoin, Ripple and Dash) using fractional integration and the Hurst component and find evidence of market inefficiency in these cryptocurrencies although they more efficiency with the passing of time. Brauneis and Mestel (2018) applied the methods used in Urquhart (2016) to examine market efficiency 73 cryptocurrencies and find evidence of Bitcoin been most market efficient cryptocurrency. Moreover, for all observed cryptocurrencies the authors compute the Garman/Klass volatility, Amihud's illiquidity measure, the Corwin/Schultz spread estimator, turnover ratio and market capitalization and find liquidity and market capitalization to positively affect efficiency whilst the spread negatively affects market efficiency. Vidal-Tomas et al. (2019) adopted a portfolio approach to measuring weak-form efficiency for 118 cryptocurrencies. Extending upon the methodologies early used by Urquhart (2016) the authors find inefficiency of cryptocurrencies and the creation of new altcoins does not improve the efficiency of the market. Noda (2021) use the generalized least squares (GLS)-based time-varying autoregressive (TV-AR) model of Noda (2016) to show that the efficiency of Bitcoin and Ethereum evolves in over time, with Bitcoin being more market efficient compared to Ethereum. Tran and Leirvik (2020) investigate weak form efficiency for 5 cryptocurrencies using the adjusted market inefficiency magnitude (AMIM) estimator of Tran and Leirvik (2019) which is a robust measure of the market inefficiency magnitude (MIM) estimator of Noda (2016). The authors find improving market efficiency in cryptocurrencies since 2013.

Besides the use of the Hurst exponent and long memory analysis in determining the informational efficiency for cryptocurrencies, there exists an even smaller scope of studies, which have more recently investigated weak-form efficiency in cryptomarkets using unit root testing procedures. For example, Latif et al. (2017) employ traditional ADF, PP and KPSS as well as the de-trend based DF-GLS and Ng-Perron tests to investigate the weak-form efficiency of Bitcoin and Litecoin returns and find that both cryptocurrency returns are stationary hence providing evidence against weak-form market efficiency. Similarly, Aggarwal (2019) applies traditional linear ADF, PP, KPSS tests as well as the structural break point tests of Perron (1989) and Zivot and Andrews (1992) and find Bitcoin returns to violate the random walk hypothesis as the series are found to be significantly stationary. Recently, Hu et al. (2019) adopt a panel approach to testing for unit roots is a panel of 31 of the top market-cap cryptocurrencies using the cross-sectional dependency tests of Chang (2002), Moon and Perron (2004), Breitung and Das (2005) and Costantini and Lupi (2013). The authors find strong evidence against weak-form market efficiency for the cryptocurrencies. And even more recently, Palamalai et al. (2020) apply parametric ADF and PP unit root tests alongside other non-parametric methods tests to examine weak-form efficiency in 10 cryptocurrencies and find evidence against the random walk hypothesis for the returns series. Nonetheless, it is well known that conventional unit root tests, such as the ADF and PP tests, used in most previous studies can be misleading if one does not account for possible nonlinearities and structural breaks in the data. Moreover, the proper modelling of structural breaks poses as a serious problem for econometricians since the number, duration, location and form of structural breaks are not known aprior (Pascalau, 2010). Our study circumvents these issues and contributes to the empirical literature by employing Fourier-based unit root testing procedures which are robust to both nonlinearities and unobserved structural breaks existing in the data.

## 4 Methodology

### 4.1 The random walk model of asset returns

Samuelson (1965) and Fama (1965) initially proposed the random walk theory as a means of testing for weak-form efficiency within a series of asset returns. The authors describe the statistical independence of asset returns (R_t_) using a general stochastic probability function defined as:

(1) Prob {R_t_ = R | R_t-1_, R_t-2_, ….} = P (R_t_ = R)

Where the first half represents the conditional probability that changes in price will take the value of R conditional on the knowledge that previous price changes took the values R_t-1_, R_t-2_ and so on whereas the second half is the unconditional probability that the price changes during time t will take on the value R (Fama, 1965). Malkiel and Fama (1970) re-specified the random walk model using one period percentage returns R_j,t+1_ i.e.

(2) f (R_j,t+1_ | Ω_t_) = f(R_j,t+1_)

Where Ω _t_ is the full historical information set from which the price at time t is derived and f is the density function for all t. In making use of the expected returns theory outlined in Mandelbrot (1966), Malkiel and Fama (1970) obtained the following statistical representation of weak form efficiency:

(3) E (R_j,t+1_ | Ω_t_) = E (R_j,t+1_)

Where E is an expectations operator. Eq. (3) illustrates that the expected return does not vary over time nor is it dependent on the historical information set, therefore analysing past information on asset values would not result in above-market profits. An alternative way of expressing Eq. (3), would be to define it as the following autoregressive (AR) random walk model of stock returns^1^:

(4) R_t_ = ρR_t-1_ + e_t_, t = 1,2,…,T and e_t_ ~ N(0, σ^2^)

From Eq. (4), the stock returns series, R_t_, is consider a random walk which confirms to the weak-form EMH only if ρ < 1 whereas if ρ = 1, then the series evolves as a stationary, predictable process which violates the weak-form EMH. Conventional random walk testing procedure such as the ADF, PP and KPSS tests commonly used amongst econometricians suffer from low power properties in distinguishing between nonlinear stationarity and unit root process as well as between random walk processes with breaks and unit root processes. To circumvent such issues, our study employs two ‘non-conventional’ unit root testing procedures, the first being the Kapetanois et al. (2003) ESTAR unit root testing procedure which is robust to asymmetries, and second the flexible Fourier form (FFF) testing procedure described in Enders and Lee (2012) which is robust to asymmetries and smooth structural breaks.

### 4.2 Testing the random walk using the KSS nonlinear unit root

In applying the KSS test to our returns series, we specify following globally stationary STAR regression:

(5) ΔR_t_ = βR_t-1_ + γR _t-1_Θ(θ; R_t-d_) + e_t_

Where e_t_ ~ iid N (0, σ^2^), γ is a smoothing parameter, c is the location parameter, d is the delay parameter and the transition function, Θ(θ; R_t-d_), used is of exponential form such that:

(6) $$\Theta \left(\text{θ;}\;{\text{R}}_{\text{t}-\text{d}}\right)=1-\exp\left(-\text{θ}R_{t-1}^{2}\right)$$

Where Θ(θ; R_t-d_):ℜ→[0,1] and is symmetrically U-shaped around zero. Kapetanois et al. (2003) consider the random walk null hypothesis a special case where β = 0 and θ = 0 in Eq. (5) whereas under the alternative hypothesis R_t_ follows an asymmetric, globally stationary process. By substitution Eq. (6) into (5) and setting β = 0 and d = 1 results in the empirically exponential smooth transition autoregressive (ESTAR) model:

(7) $$\text{Δ}{\text{R}}_{\text{t}}=\text{γ}{\text{R}}_{\text{t}-1}\left\{1-\exp\left(-\text{θ}R_{t-1}^{2}\right)\right\}+{\text{e}}_{\text{t}}$$

Since γ is unidentified in Eq. (7), Kapetanois et al. (2003) propose the application of a first-order Taylor expansion to Eq. (7) around γ = 0 and obtain the following nonlinear random walk testing model:

(8) $$\text{Δ}{\text{R}}_{\text{t}}=\delta R_{t-1}^{3}+\text{error}$$

In considering a more generalized form of regression (8) where the errors are serially correlated, Eq. (9) can be extended to included lags on the differenced time series as follows:

(9) $$\text{ΔRt}=\delta R_{t-i}^{3}+\sum_{j=1}^{p}\rho_{i}\Delta R_{t-i}+e_{t}$$

From Eqs. (8) and (9), the null hypothesis of a random walk is tested as:

(10) H_0_: δ = 0

Against the alternative of a stationary process i.e.

(11) H_1_: δ < 0

And the test statistic evaluating these hypotheses is defined as:

(12) $$t_{KSS}=\frac{\hat{\delta }}{se\left(\hat{\delta }\right)}$$

Where $\hat{\delta }$ is the OLS estimator of δ and $se\left(\hat{\delta }\right)$ is the standard error of $\hat{\delta }$. Since the t_KSS_ statistic does not follow an asymptotic standard normal distribution, Kapetanios et al. (2003) derive critical values for the test statistics for the test performed on raw time series, de-meaned data (i.e. z_t_ = x_t_ – ${\bar{x}}_{t}$) and de-trended data (i.e. z_t_ = x_t_ – $\hat{\mu }$– $\hat{\delta }t$) where ${\bar{x}}_{t}$ is the sample mean and $\hat{\mu }$ and $\hat{\delta }t$ are the OLS estimates of μ and δ, respectively.

### 4.3 Testing the random walk via flexible fourier functions

Enders and Lee (2012) consider a simple modification of the Dickey-Fuller test in which a time-dependent deterministic term, d(t), is added to the test regression i.e.

(13) R_t_ = d(t) + ρR_t-1_ + e_t_ e _t_~ N(0, σ^2^)

Assuming that the functional form of d(t) is known, the unit root null hypothesis of p = 1 can be tested by approximating d(t) with the following single frequency Fourier function/equation:

(14) d(t) = α_0_ + α_sin_ sin(2πkt/T) + α_cos_ cos(2πkt/T)

Where k is the single frequency component and measure the amplitude and displacement of the sinusoidal component of d(t). Using our cryptocurrency return series (R_t_), we model the underlying data generating process as the following FFF function:

(15) R_t_ = α_0_ + α_sin_ sin(2πkt/T) + α_cos_ cos(2πkt/T) + ε_t_; k ≤ T/2

(16) e_t_ = ρe_t-1_ + u_t_

To test the random walk null hypothesis of ρ = 1 against the stationary alternative ρ < 1, we follow Enders and Lee (2012) by employing a langrage Multiplier (LM) procedure to the following first differences econometric regression i.e.

(17) ΔR_t_ = δ_0_ + δ_1_ Δsin(2πkt/T) + δ_2_ Δcos(2πkt/T) + ε_t_

And by using the estimated coefficients $\tilde{\delta }$_0,_ $\tilde{\delta }$_1_ and $\tilde{\delta }$_2_ from Eq. (17) we construct a de-trended series as follows:

(18) $${\tilde{S}}_{t}={\text{R}}_{\text{t}}-\tilde{\psi }-{\tilde{\delta }}_{0}\text{t}-{\tilde{\delta }}_{1}\text{Δsin}\left(2\text{πkt/T}\right)-{\tilde{\delta }}_{2}\text{Δcos}\left(2\text{πkt/T}\right),\text{t}=2,\ldots ,T$$

Where $\tilde{\psi }$ = R_1_ - $\tilde{\delta }$_1_ - $\tilde{\delta }$_1_Δsin (2πkt/T) - $\tilde{\delta }$_2_Δcos (2πkt/T). In subtracting $\tilde{\psi }$ from R_t_ we then obtain $\tilde{S}$_1_ = 0. Using the de-trended series, $\tilde{S}$_t_, we formulate our econometric random walk test regression as:

(19) $$\text{Δ}{\text{R}}_{\text{t}}=\varphi {\tilde{S}}_{t-1}+{\text{d}}_{\text{0}}+{\text{d}}_{1}\text{Δsin}\left(2\text{πkt/T}\right)-{\text{d}}_{2}\text{Δcos}\left(2\text{πkt/T}\right)+{\text{ε}}_{\text{t}}$$

And in adding lags to the first differences of $\tilde{S}$_t_ regression (19) to remove possible serial correlation, produces the following augment FFF-based unit testing regression:

(20) $$\text{Δ}{\text{R}}_{\text{t}}=\varphi {\tilde{S}}_{t-1}+{\text{d}}_{\text{0}}+\sum_{j=1}^{p}\rho_{j}\text{Δ}{\tilde{S}}_{t-j}+{\text{d}}_{1}\text{Δsin}\left(2\text{πkt/T}\right)-{\text{d}}_{2}\text{Δcos}\left(2\text{πkt/T}\right)+{\text{ε}}_{\text{t}}$$

From Eqs. (19) and (20) the random walk null hypothesis is tested as the t-statistic (τ_DF_t_) for the following null hypothesis, H_0_: φ = 0 and the empirical procedure is then practically carried out in the following four-steps:

Step 1 Perform a two-dimensional grid search for combinations of all integers of k bounded between 1 ≤ k ≤ 5 and lags lengths bounded between 0 < j < 20. The optimal values of [k∗, j∗] are those associated with the regression that yields the lowest sum of squared residuals (SSR).

Step 2 Perform a test of linearity using the F-statistics to test the null hypothesis of d_1_ = d_2_ = 0. Since the distribution of the F-statistic is non-standard, we rely on the critical values tabulated in Enders and Lee (2012).

Step 3 Evaluate the estimated regression for serial correlation using the traditional Durbin Watson (DW) statistic.

Step 4 Compare the τ_LM_ statistic against the critical values of reported in Enders and Lee (2012) for different sample sizes.

## 5 Data and results

### 5.1 Empirical data and descriptive statistics

The sample data was sourced from Coingecko (https://www.coingecko.com/en), a cryptocurrency exchange platform, and we specifically collect daily and weekly trading values for the five cryptocurrencies, namely Bitcoin (BTC), Ethereum (ETH), Litecoin, Bitcoin Cash (BTCC) and Ripple (RIP). Table 1 provides a summary of these cryptocurrencies in terms of founder, date found, market capitalization in US dollars in 2018, our data source as well as the sampled time period collected for each cryptocurrency examined in our empirical analysis:

**Table 1:** Data sources and cryptocurrency overview.

Cryptocurrency	Year founded	Developer	Market capitalization (US$) (2018)	Data source	Sample data period
Bitcoin	2009	Satoshi Nakamoto	108,955,587,484	Coingecko	09/01/2009–31/10/2019
Ethereum	2015	Vitalik Buterin	17,627,290,682	Coingecko	30/06/2015–31/10/2019
Bitcoin cash	2017	Amaury Sechet	7,324,564,447	Coingecko	01/08/2017–31/10/2019
Litecoin	2011	Charles Lee	2,867,248,642	Coingecko	13/10/2011–31/10/2019
Ripple	2016	Ripple Labs	10,466,673,095	Coingecko	03/06/2016–31/10/2019

The daily and weekly returns, R_t_, are computed using the following continuously compounded returns formula:

(21) $${\text{R}}_{\text{t}}=100\times \ln\frac{P_{t}}{P_{t-1}}$$

**Figure 1:**
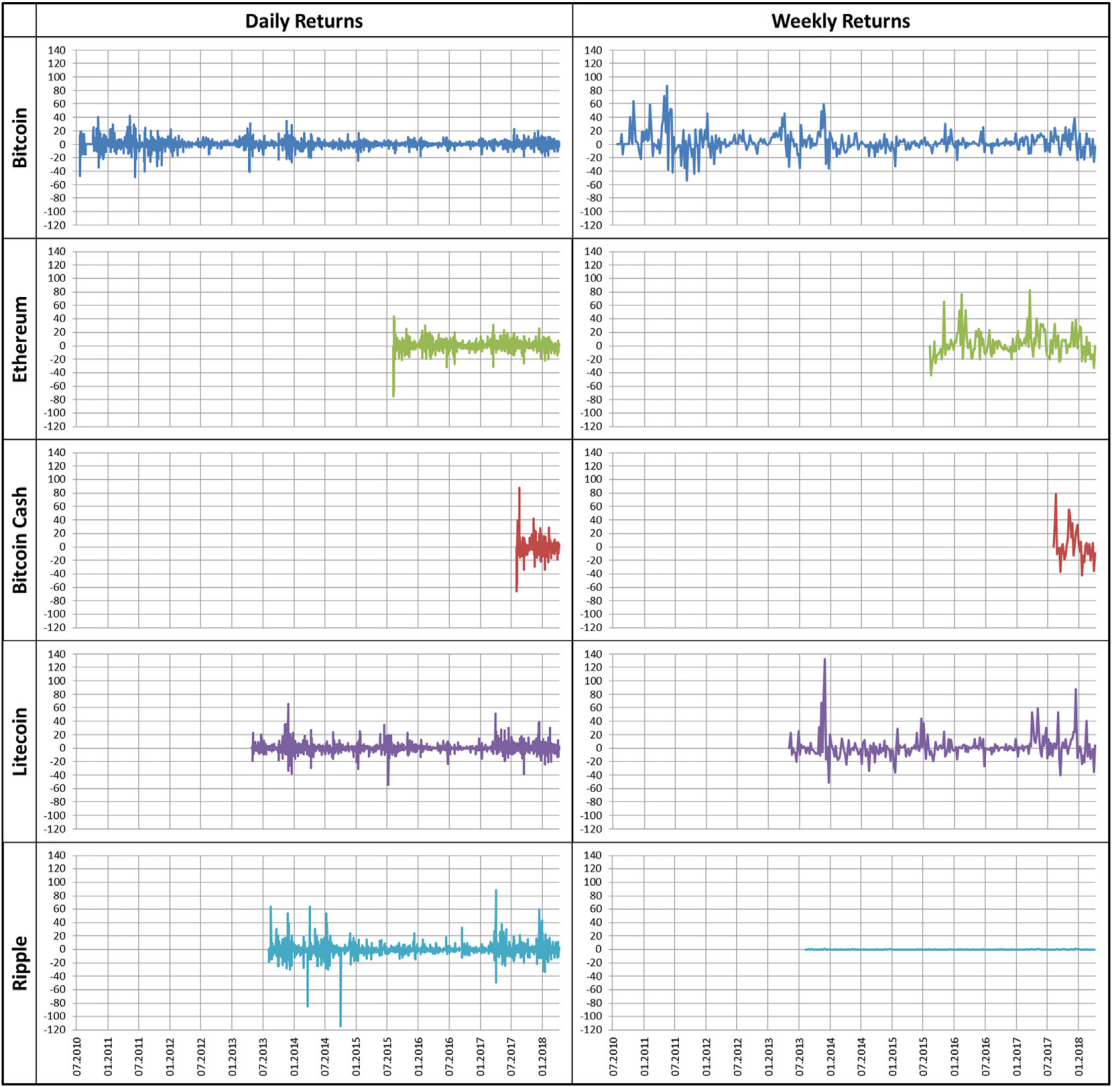
Daily and weekly returns for all cryptocurrencies

Figure 1 below presents the time series plots of the log-returns form of our cryptocurrencies for daily and weekly frequencies. Table 2 presents summary statistics for the returns series which are difficult to dissect solely through visual appreciation of the time series plots. In terms of financial performance of the individual asset returns, ETH outperforms BTC attracting higher risk investors as its high yields are commensurate with the highly volatile nature of its returns. LTC and RIP are moderate-return assets with lower risk than BTC and ETH but higher returns than BTX. The relatively new BTX, which joined the cryptocurrency market in 2017, has more strides to make to establish itself in the virtual currency market as it attracts a lot of risk which is associated with suboptimal returns compared to the more established digital currencies. A noteworthy statistic which was manually computed for the purpose of making comparisons across the different series was the coefficient of variation (CV), which is indicative of the risk-return trade-off that an investor is offered when evaluating an asset for their portfolio. The computed CV supports this analysis as it shows the most profitable cryptocurrencies with higher units of risk being commensurate with the anticipated return.

**Table 2:** Summary statistics.

	Skew	Kurt	Jarque-Bera	p-value	Mean	St. dev	Average CV	Max R	Min R
BTC-D	-0.42	15.33	17715.35	0,00∗∗∗	0.41	5.89	14.38	42.46	-49.15
ETH-D	-1.19	19.09	10766.34	0,00∗∗∗	0.50	7.89	15.56	43.98	-75.51
BTX-D	0.79	16.93	2055.64	0,00∗∗∗	-0.07	12.39	-169.45	87.47	-65.49
LTC-D	1.26	20.57	23726.89	0,00∗∗∗	0.18	6.73	37.05	65.88	-54.72
RIP-D	-0.02	35.28	73959.24	0,00∗∗∗	0.25	9.04	34.77	88.13	-114.51
BTC-W	0.83	7.61	398.50	0,00∗∗∗	2.97	15.89	5.35	86.57	-54.09
ETH-W	1.16	5.57	69.98	0,00∗∗∗	3.54	19.94	5.65	82.75	-43.85
BTX-W	0.85	4.07	5.92	0,05∗	2.90	25.40	8.75	78.23	-42.43
LTC-W	2.59	17.29	2476.74	0,00∗∗∗	1.46	18.03	12.34	132.81	-51.47
RIP-W	1.90	9.47	568.64	0,00∗∗∗	0.019	0.23	11.62	1.22	-0.70

Notes: “∗∗∗”, ‘∗∗’, ‘∗’ denote 1%, 5%, 10% critical levels, respectively.

Besides the initial moments of mean and standard deviation (St.Dev), it is essential to also describe the higher moments of skewness and kurtosis. The former describes lack of symmetry about the mean akin to the normal distribution whereas the latter focuses on fatness of the tails and peakedness at the mean, a significant characteristic of financial asset returns. All observed time series appear to be positively skewed exhibiting longer upper tails than lower tails pointing to the result a substantial portion of the returns tend to be more positive. The kurtosis of the of the asset returns generally appears to be in excess of 3, meaning their distribution is mostly characterised as leptokurtic which is to be expected with financial time series. The reported Jarque-Bera (JB) statistics for all the daily and weekly cryptocurrencies returns series reject normality at all levels of significance with sole exception of the BTX weekly series which only rejects the normality hypothesis at a 10% critical level. More of the results of non-parametric test of Broock et al. (1996) for independence and identical distribution in the cryptocurrency returns, as reported in Table 3, fails to confirm that any of the series is i.i.d., which is a violation of the weak-form market efficiency. Nonetheless, the absence of normality in the observed cryptocurrencies returns series is not atypical to characteristics of finical time series and strengthens our case for possible existing asymmetries and structural breaks existing within the series.

**Table 3:** BDS test for linear dependence (p-values).

	2	3	4	5	6
BTC-D	0.0000∗∗∗	0.0000∗∗∗	0.0000∗∗∗	0.0000∗∗∗	0.0000∗∗∗
ETH-D	0.0000∗∗∗	0.0000∗∗∗	0.0000∗∗∗	0.0000∗∗∗	0.0000∗∗∗
BTX-D	0.0000∗∗∗	0.0000∗∗∗	0.0000∗∗∗	0.0000∗∗∗	0.0000∗∗∗
LTC-D	0.0000∗∗∗	0.0000∗∗∗	0.0000∗∗∗	0.0000∗∗∗	0.0000∗∗∗
RIP-D	0.0000∗∗∗	0.0000∗∗∗	0.0000∗∗∗	0.0000∗∗∗	0.0000∗∗∗
BTC-W	0.0000∗∗∗	0.0000∗∗∗	0.0000∗∗∗	0.0000∗∗∗	0.0000∗∗∗
ETH-W	0.0000∗∗∗	0.0000∗∗∗	0.0000∗∗∗	0.0000∗∗∗	0.0000∗∗∗
BTX-W	0.0000∗∗∗	0.0000∗∗∗	0.0000∗∗∗	0.0000∗∗∗	0.0000∗∗∗
LTC-W	0.0000∗∗∗	0.0000∗∗∗	0.0000∗∗∗	0.0000∗∗∗	0.0000∗∗∗
RIP-W	0.0000∗∗∗	0.0000∗∗∗	0.0000∗∗∗	0.0000∗∗∗	0.0000∗∗∗

Notes: “∗∗∗”, ‘∗∗’, ‘∗’ denote 1%, 5%, 10% critical levels, respectively.

### 5.2 Preliminary unit root tests

For comparison sake, we begin our empirical analysis by providing estimates from the first generation unit root tests, that is, unit root testing procedures which neither take into consideration asymmetries or structural breaks in the data generating process. Table 4 presents the results from the conventional ADF, PP and KPSS integration tests performed on the cryptocurrency returns. The results obtained from both ADF and PP unit root tests reject the random walk null hypothesis at critical levels of at least 5 percent for all cryptocurrencies regardless of whether the tests are performed with a drift only or inclusive of a trend, or whether the tests are performed on daily or weekly series. Note that this evidence against weak form market efficiency in the cryptomarkets has been previously established in the works of Latif et al. (2017) and Aggarwal (2019) who use similar ADF and PP unit root testing procedures albeit on weekly series. Moreover, these results are also consistent with findings obtained from the cross-sectional dependent, panel unit root tests employed by Hu et al. (2019). Conversely, the outcomes from the KPSS test, which examines the null hypothesis of a stationary returns process, were not significantly different from those of the ADF and PP tests, with the exception of ETH and BTX daily (D) returns, which rejected the null of stationarity, at critical values of 10 percent and 5 percent respectively, in favour of a random walk in the cryptocurrency returns.

**Table 4:** ADF, PP and KPSS test results.

	Lag (ADF)	ADF	PP	KPSS
BTC-D-I	26	-6.54274∗∗∗	-52.1545∗∗∗	0.281793
BTC-D-TI	26	-6.7734∗∗∗	-52.1322∗∗∗	0.106746
ETH-D-I	21	-4.61274∗∗∗	-32.5997∗∗∗	0.164461
ETH-D-TI	21	-4.61499∗∗∗	-32.5951∗∗∗	0.152466∗∗
BTX-D-I	15	-3.42499∗∗	-14.813∗∗∗	0.151124
BTX-D-TI	15	-3.41896∗	-14.984∗∗∗	0.122985∗
LTC-D-I	24	-7.18248∗∗∗	-40.1168∗∗∗	0.227335
LTC-D-TI	24	-7.21897∗∗∗	-40.1062∗∗∗	0.069515
RIP-D-I	8	-11.1363∗∗∗	-37.8853∗∗∗	0.130309
RIP-D-TI	8	-11.1642∗∗∗	-37.8813∗∗∗	0.050239
BTC-W-I	13	-3.7382∗∗∗	-18.3494∗∗∗	0.221428
BTC-W-TI	5	-6.02975∗∗∗	-18.3725∗∗∗	0.079318
ETH-W-I	2	-4.9616∗∗∗	-10.7566∗∗∗	0.104396
ETH-W-TI	2	-4.92133∗∗∗	-10.718∗∗∗	0.096987
BTX-W-I	1	-3.83214∗∗∗	-4.14457∗∗∗	0.279491
BTX-W-TI	1	-3.71515∗∗	-4.39042∗∗∗	0.072638
LTC-W-I	4	-5.87585∗∗∗	-14.0398∗∗∗	0.186594
LTC-W-TI	4	-5.90551∗∗∗	-14.0396∗∗∗	0.080387
RIP-W-I	5	-5.47356∗∗∗	-12.2411∗∗∗	0.141466
RIP-W-TI	5	-5.57857∗∗∗	-12.1808∗∗∗	0.056625

Notes: The modified SC criterion is used to determine optimal lag length of the ADF tests.

W – Weekly series, D – Daily series, I – intercept, IT – trend and intercept.

“∗∗∗”, ‘∗∗’, ‘∗’ denote 1%, 5%, 10% critical levels, respectively.

However, it is well-known that conventional unit root tests suffer from low power properties and size distortions problems in distinguishing between unit root and close-to-unit root processes. Therefore, we further supplement our preliminary unit root tests by estimating the modified Dickey-Fuller generalised least squares (DF-GLS) statistic of Elliot, Rothenberg and Stock (1996) and Ng and Perron (2001) which are considered more powerful in the estimation of parameters in a deterministic autoregressive process. Findings from these tests are presented in Table 5, and as can be easily observed, the findings are mixed. For the DF-GLS tests, BTC, ETH and BTX daily (D) returns series favoured non-stationarity whereas the LTC and RIP series rejected non-stationarity. For the weekly (W) series, BTC, BTX and RIP rejected the null of a unit root whereas ETH and LTC failed to reject the unit root null hypothesis. The Ng-Perron results also comprised mixed results with most findings coinciding with the DF-GLS test results. For the Ng-Perron tests, RIP daily returns series as well as BTX weekly returns series both failed to reject the null when the inclusion of a trend. Notably the inconclusiveness of our DF-GLS and Ng-Perron tests in evaluating random walk behaviour for the Bitcoin returns has been previously reported in an earlier study by Latif et al. (2017) and Aggarwal (2019) and Palamalai et al. (2020) albeit strictly for daily Bitcoin returns.

**Table 5:** DF-GLS and Ng-Perron test results.

	DF-GLS		Ng-Perron							
	t-statistic	Lag	MZa	Lag	MZt	Lag	MSB	Lag	MPT	Lag
BTC-D-I	-1.01879	21	-1.04267	21	-0.61058	21	0.58559	21	18.7965	21
BTC-D-TI	-0.22046	21	-1.00575	21	-0.47471	21	0.472	21	48.602	21
ETH-D-I	-0.16293	18	0.45739	18	1.71939	18	3.75912	18	789.805	18
ETH-D-TI	-2.07776	10	-0.25683	10	-0.24115	10	0.93893	10	168.597	10
BTX-D-I	-0.33326	7	0.14644	7	0.19034	7	1.29977	7	93.2028	7
BTX-D-TI	-1.61475	6	-0.93585	6	-0.59586	6	0.63671	6	77.3669	6
LTC-D-I	-5.8587∗∗∗	24	-21.4325∗∗∗	24	-3.26723∗∗∗	24	0.15244∗∗∗	24	1.16577∗∗∗	24
LTC-D-TI	-6.56342∗∗∗	24	-34.9695∗∗∗	24	-4.18102∗∗∗	24	0.11956∗∗∗	24	2.60847∗∗∗	24
RIP-D-I	-2.95345∗∗∗	24	-7.72658∗	24	-1.96056∗	24	0.25374∗	24	3.19016∗	24
RIP-D-TI	-4.16467∗∗∗	24	-12.6136	24	-2.51115	24	0.19908	24	7.22542	24
BTC-W-I	-3.56791∗∗∗	13	-32.4704∗∗∗	13	-3.98954∗∗∗	13	0.12311∗∗∗	13	0.86637∗∗∗	13
BTC-W-TI	-3.24091∗∗	13	-20.7892∗∗	13	-3.20671∗∗	13	0.15425∗∗	13	4.49151∗∗	13
ETH-W-I	-1.54738	2	-4.35397	2	-1.42857	2	0.32811	2	5.70978	2
ETH-W-TI	-1.40319	6	-4.15202	6	-1.40266	6	0.33782	6	21.5522	6
BTX-W-I	-2.95838∗∗∗	1	-8.39868∗∗	1	-2.01092∗∗	1	0.23943∗	1	3.06096∗∗	1
BTX-W-TI	-3.47647∗∗	1	-10.8567	1	-2.32878	1	0.2145	1	8.39882	1
LTC-W-I	-1.70072∗	8	-4.52673	8	-1.4767	8	0.32622	8	5.46934	8
LTC-W-TI	-2.21926	15	-4.04674	15	-1.40779	15	0.34788	15	22.3526	15
RIP-W-I	-1.84764∗	5	-5.35983	5	-1.602	5	0.29889	5	4.6727	5
RIP-W-TI	-3.70443∗∗∗	8	-9.11106	8	-2.10697	8	0.23125	8	10.1148	8

Notes: The modified SC criterion is used to determine optimal lag length of the tests.

W – Weekly series, D – Daily series, I – intercept, IT – trend and intercept.

“∗∗∗”, ‘∗∗’, ‘∗’ denote 1%, 5%, 10% critical levels, respectively.

### 5.3 Second generation unit root tests

The next phase in our empirical process is to determine whether accounting for nonlinearities would change the outlook on the inefficiency of cryptocurrencies generally assumed by the results of our conventional unit root tests. As argued by Kapetanois et al. (2003), transaction costs and other frictions in financial assets markets are likely to lead to nonlinear equilibrium adjustments which linear unit root tests would exert low power in differentiating from unit root processes. Table 6 presents the empirical results for the KSS nonlinear test performed on our cryptocurrency returns. Note that these tests have been performed on raw, de-meaned and de-trended transformations of the returns series for daily and weekly data. The KSS tests statistics obtained from the raw data manage to reject the random walk hypothesis at all critical levels for 9 out of 15 cases with the random walk hypothesis failing to be rejected for the raw, de-meaned and de-trended BTC daily series as well as for the raw, de-meaned and de-trended RIP weekly series. Note that these findings are comparable to the findings obtained from the conventional ADF, PP and KPSS testing procedures but are in stark contrast to the outcomes of the DF-GLS and Ng-Perron tests.

**Table 6:** KSS test results.

Daily Data	Lag	t statistic	Weekly Data	Lag	t statistic
BTC-R	13	-9.24792∗∗∗	BTC-R	3	-6.38122∗∗∗
ETH-R	11	-5.61394∗∗∗	ETH-R	2	-3.85103∗∗∗
BTX-R	15	-0.96025	BTX-R	1	-3.67979∗∗∗
LTC-R	14	-3.1336∗∗∗	LTC-R	4	-4.71456∗∗∗
RIP-R	14	-7.11717∗∗∗	RIP-R	8	-1.41891
BTC-DM	13	-9.19059∗∗∗	BTC-DM	3	-6.72606∗∗∗
ETH-DM	11	-5.77731∗∗∗	ETH-DM	2	-3.8225∗∗∗
BTX-DM	15	-0.95672	BTX-DM	1	-3.75798∗∗∗
LTC-DM	14	-3.15943∗∗	LTC-DM	4	-4.72947∗∗∗
RIP-DM	14	-7.07004∗∗∗	RIP-DM	8	-1.43298
BTC-DT	13	-9.13999∗∗∗	BTC-DT	3	-6.90533∗∗∗
ETH-DT	11	-5.78869∗∗∗	ETH-DT	2	-3.85155∗∗
BTX-DT	15	-0.95699	BTX-DT	1	-3.78479∗∗
LTC-DT	14	-3.14054∗	LTC-DT	4	-4.59533∗∗∗
RIP-DT	14	-7.05642∗∗∗	RIP-DT	8	-1.43348

Notes: The modified SC criterion is used to determine optimal lag length of the tests.

R – Raw series, DM – De-meaned series, DT – De-tended series.

“∗∗∗”, ‘∗∗’, ‘∗’ denote 1%, 5%, 10% critical levels, respectively.

### 5.4 FFF-unit root test results

In the final stage of our empirical analysis, we carry out the LM-type Fourier unit root testing procedure described in Enders and Lee (2012) on the cryptocurrency returns series and record our findings in Table 7. Note that we carry out this testing procedure in four phases. Firstly, we performed a two-dimensional grid search for the optimal combinations of the length of Fourier function frequencies, k∗, and the lag length of the first series of the ‘FFF de-trended series, j∗. The optimal combinations of k∗ and j∗ are obtained as those which simultaneously minimizes the RSS and these estimates are reported in columns (1) and (2) of Table 7, respectively. Note that the optimal frequencies, k∗, were found to be either one or two, which is in line with the findings of other authors including the Enders and Lee (2012), Becker et al. (2006), Pascalau (2010) and Rodrigues and Taylor (2012). Secondly, to ensure that the optimal lag length, j∗, removes all possible serial correlation we estimate the DW statistic and obtained statistics reported in column (3) of Table 6 indicate that our FFF-based unit root regression estimates are devoid of an autocorrelation. Note that for the BTX, LTC and RIP weekly series, we did not include lags as there was no evidence of serial correlation present (indicated by the Durbin Watson statistic). In fact, including lags resulted in a less than optimal Durbin Watson (DW) statistic, therefore the test regression that was estimated for the BTX, LTC and RIP weekly series was estimated with no lags.

**Table 7:** LM test results with the flexible Fourier form.

	frequency	Lag	F statistic	t statistic	DW	T
BTC-D	1	13	123.5927∗∗∗	-0.65617	2.007	2826
ETH-D	1	10	61.1041∗∗∗	-1.7428	2.014	976
BTX-D	1	15	15.34289∗∗∗	-2.37522	1.973	251
LTC-D	1	5	201.7307∗∗∗	-13.7081∗∗∗	2.01	1806
RIP-D	1	14	72.8367∗∗∗	-1.84738	2.01	1706
BTC-W	1	3	43.39032∗∗∗	-4.15214∗∗	2.03	403
ETH-W	2	1	24.73095∗∗∗	-5.00412∗∗∗	2.11	139
BTX-W	2	no lag	9.70371∗∗∗	-5.26262∗∗∗	1.905	35
LTC-W	1	no lag	63.52684∗∗∗	-13.8044∗∗∗	2.006	257
RIP-W	1	no lag	24.82248∗∗∗	-8.62497∗∗∗	2.04	243

Notes: The modified SC criterion is used to determine optimal lag length of the tests.

W – Weekly series, D – Daily series.

“∗∗∗”, ‘∗∗’, ‘∗’ denote 1%, 5%, 10% critical levels, respectively.

Thirdly, we test our regressions for linearity using the LM test described in Enders and Lee (2012) and report the obtained F-statistics in column (4) of Table 6. Critical values reported in Enders and Lee (2012) were adopted at T = 500 for all daily returns with the exception of BTX which had fewer observations hence utilising critical values at T = 100. Similarly, with respect to the weekly data, the critical values utilised were at T = 500 with the exception of BTX which we relied on utilising critical values at T = 100. In performing our linearity tests, we find all cryptocurrencies rejected the null hypothesis in favour of nonlinearity, regardless of whether daily or weekly series are used. Lastly, we provide estimates of our τ_LM_ statistic for all cryptocurrency series with the results reported in column (5) of Table 7. Interestingly enough, before adding lags to the regressions we found that all currencies rejected the null in favour of stationarity and yet the test statistics are significant reduced in absolute value for most of the daily series subsequent to the addition of lags except for the LTC daily series as shown in Table 7. Note that most cryptocurrencies in the daily series failed to reject the null of a unit root except for the LTC returns series. This notwithstanding, all cryptocurrency returns in the weekly series rejected the null of non-stationarity in favour of stationarity. These latter findings bear close similarity to those obtained from the conventional linear unit root test results as well as the outcomes of the KSS test where most series failed to provide formal evidence of weak-form market efficiency.

## 6 Conclusions

The exponential growth of the peer-to-peer digital currency trading in the aftermath of 2007–2008 sub-prime crisis has attracted increasing interest over whether these cryptocurrency markets are informational efficient. In our study, we rely on the random walk model of stock returns to investigate the weak-form market efficiency hypothesis for five of the most dominant cryptocurrencies (Bitcoin, Ethereum, Litecoin, Bitcoin Cash and Ripple) by employing a battery of random walk tests ranging from conventional unit root tests to integration tests which account for nonlinearities and unobserved structural breaks. Moreover, we examine the impact of return frequency intervals on the market efficiency for the case of daily and weekly return series. The outcomes from our empirical endeavours can be summarized in two main findings.

Firstly, in applying traditional unit root tests such as the ADF, PP and KPSS tests as well as the KSS nonlinear test provided strong evidence against weak-form informational efficiency for all observed cryptocurrencies regardless of whether daily or weekly returns are employed. Secondly, in relying on the more powerful FFF-based unit root testing procedure which is robust to both asymmetries and unobserved structural breaks, our findings show discrepancies with respect to the frequency intervals of the cryptocurrency returns. In particular, we find that, with the exception of Litecoin, daily series are generally market efficient whilst all weekly returns are informationally inefficient. In other words, ‘less-noisy’ weekly trading systems, as opposed to daily trading systems, can be used to generate abnormal profits in cryptocurrency markets. Therefore, high frequency traders who use algorithmic trading programmes to make multiple high-speed trades on an intra-day basis are unlikely to beat the market whilst those using weekly-based strategies face a better chance of beating the market. Our findings hence imply that the cryptocurrencies are not market efficient enough to be considered as a more formal exchange system and more regulatory intervention is required in cryptocurrency markets.

## Declarations

### Author contribution statement

Andrew Phiri and Natalya Apopo: Conceived and designed the experiments; Performed the experiments; Analyzed and interpreted the data; Contributed reagents, materials, analysis tools or data; Wrote the paper.

### Funding statement

This research did not receive any specific grant from funding agencies in the public, commercial, or not-for-profit sectors.

### Data availability statement

The authors do not have permission to share data.

### Declaration of interests statement

The authors declare no conflict of interest.

### Additional information

No additional information is available for this paper.

## Footnotes

1 It is important to note that whilst there is no strict connection between efficient market hypothesis and the random walks (see LeRoy (1973) and Lucas (1978)) most empirical studies are focused on the weak-form efficiency to examine whether future prices are purely unpredictable based on the asset's price history.
